# Chemical doping of MoS_2_ multilayer by p-toluene sulfonic acid

**DOI:** 10.1088/1468-6996/16/3/035009

**Published:** 2015-06-04

**Authors:** Shaista Andleeb, Arun Kumar Singh, Jonghwa Eom

**Affiliations:** 1 Department of Physics and Graphene Research Institute, Sejong University, Seoul 143-747, Korea; 2 Department of Physics, Motilal Nehru National Institute of Technology, Allahabad-211004, India

**Keywords:** molybdenum disulfide, field-effect transistor, p-toluene sulfonic acid, doping

## Abstract

We report the tailoring of the electrical properties of mechanically exfoliated multilayer (ML) molybdenum disulfide (MoS_2_) by chemical doping. Electrical charge transport and Raman spectroscopy measurements revealed that the p-toluene sulfonic acid (PTSA) imposes n-doping in ML MoS_2_. The shift of threshold voltage for ML MoS_2_ transistor was analyzed as a function of reaction time. The threshold voltage shifted toward more negative gate voltages with increasing reaction time, which indicates an n-type doping effect. The shift of the Raman peak positions was also analyzed as a function of reaction time. PTSA treatment improved the field-effect mobility by a factor of ~4 without degrading the electrical characteristics of MoS_2_ devices.

## 1. Introduction

Over the last several years, two-dimensional (2D) semiconducting transition-metal dichalcogenide (TMD) has attracted increased research attention because of its emerging electrical and optical properties and great potential in practical applications [1, 2]. Graphene is a representative 2D material that has a conical Dirac spectrum of energy states without a band gap, which results in many interesting physical properties as well as stimulating applications [3–6]. However, the gapless band structure of graphene makes it unsuitable as an electronic material in logic circuits. Molybdenum disulfide (MoS_2_), a layered TMD, has emerged as a feasible alternative to graphene because it has a moderate energy gap with mechanical flexibility, chemical and thermal stability, and absence of dangling bonds [7–14]. A layer of MoS_2_ consists of a molybdenum monolayer sandwiched between two sulfur monolayers. The strong intralayer bonds and weak interlayer van der Waals forces make it possible to exfoliate individual MoS_2_ layers. MoS_2_ crystals can also be obtained in a large scale by chemical exfoliation and chemical vapor deposition (CVD) techniques [15].

MoS_2_-based electronic and optoelectronic devices such as field-effect transistors (FETs), integrated circuits, solar cells, photodetectors, memory devices, chemical and biosensors, supercapacitors, and photocatalyzed hydrogen evolution reactors have been successfully fabricated [10, 14–17]. FET is a basic and important application of semiconducting TMD materials. Several issues, including reducing the contact resistance, using different substrates, and depositing high-k materials as the top gate, have been focused on to improve the performance of MoS_2_ FET [18–22]. Given the ultrathin structure of MoS_2_ layers, doping of this material has yet to be fully developed. Traditional methods of doping such as ion implantation are not suitable for MoS_2_ thin layer; hence, alternative approaches, such as chemical and molecular doping, must be explored.

Chemical doping has been used to investigate the surface charge transfer between dopant molecules and MoS_2_ layer [23]. The charge transfer between dopant and host material modulates the Fermi level and results in the modification of the optical and electrical properties of 2D materials. Chemical doping of MoS_2_ nanoflakes with solution-based dopants, gases, metal nanoparticles, or self-assembled monolayers has already been reported [24–28]. Mouri *et al* reported the tunable photoluminescence of monolayer MoS_2_ flakes by n-type and p-type dopants in chemical solution [25]. Fang *et al* reported a degenerate n-doping of MoS_2_ by potassium metal ions and the significant change in the electron density of MoS_2_ nanoflakes [26]. Gold nanoparticles were used by Shi *et al* to effectively decorate MoS_2_ layers via wet chemical method [27]. They found that gold nanoparticles impose p-doping to the MoS_2_ transistors. Li *et al* studied the carrier doping of MoS_2_ nanoflakes by functional self-assembled monolayers with different dipole moments [28]. Our group also demonstrated ultraviolet light-induced reversible and stable charge carrier modulation in single-layer, bilayer and multilayer (ML) MoS_2_ nanosheets with a combination of N_2_ and O_2_ gases [29].

Here, we report the tailoring of the electronic properties of ML MoS_2_ by p-toluene sulfonic acid (PTSA) molecular doping. PTSA is a well-known dopant for conducting polymers and is highly soluble in water. The charge transport measurements and Raman spectroscopy revealed that PTSA molecule imposes n-doping in ML MoS_2_. The threshold voltages shifted toward more negative gate voltages after exposure of PTSA molecules for different periods of time, revealing the n-doping in ML MoS_2_. The charge carrier density and field-effect mobility were also estimated and found to be significantly improved after PTSA treatment. Results indicate that chemical modification is a simple approach to tailor the electrical properties of few-layered MoS_2_ while maintaining its important electrical characteristics.

## 2. Experimental details

### 2.1. Sample preparation

ML MoS_2_ film was mechanically exfoliated from bulk crystals of molybdenite (SPI Supplies, USA) by the scotch tape method and then transferred to a 300 nm thick highly p-doped SiO_2_/Si substrate. Optical microscopy, Raman spectroscopy, and atomic force microscopy (AFM) aided in identifying the number of layers of the MoS_2_ flakes. The ML MoS_2_ had seven to eight monolayers. Raman spectra were obtained with a Renishaw microspectrometer with 514 nm laser wavelength at room temperature. The laser power was kept at ≈1.0 mW to avoid local heating and defect introduction by the laser.

### 2.2. Device fabrication and measurements

Photolithography technique was used to make large patterned electrodes (Cr/Au of 6/30 nm thicknesss) for ML MoS_2_ devices. As the final process, Cr/Au (10/80 nm) ohmic contacts were patterned by e-beam lithography. The channel length of transistors was kept almost same (~2.8 *μ*m) for all devices. The devices were annealed in a tube furnace at 200 °C in a flow of 100 sccm Ar and 10 sccm H_2_ for 4 h to remove lithography resist residue and minimize the contact resistance of the devices. Electrical characterizations of the devices were performed using Keithley 2400 and Picometer 6485 instruments by two-probe measurements at room temperature in vacuum.

### 2.3. PTSA doping and characterizations

Electrical charge transport measurements and Raman spectroscopy were used to characterize the pristine ML MoS_2_ film. The same device was then doped with PTSA for different periods of time, and the effect of PTSA doping was investigated by charge transport measurements and Raman spectroscopy. The PTSA monohydrate (ACS reagent, ≥ 98.5%, Aldrich) was dissolved in deionized water to make a PTSA solution with 0.1 M concentration. The ML MoS_2_ film on the Si/SiO_2_ substrate was soaked in the PTSA solution for certain periods of time and blow-dried by nitrogen. The sample was then placed in a vacuum desiccator for 1 d to completely dry. The procedure described in our previous papers was followed [30, 31].

## 3. Results and discussion

**Figure 1.**
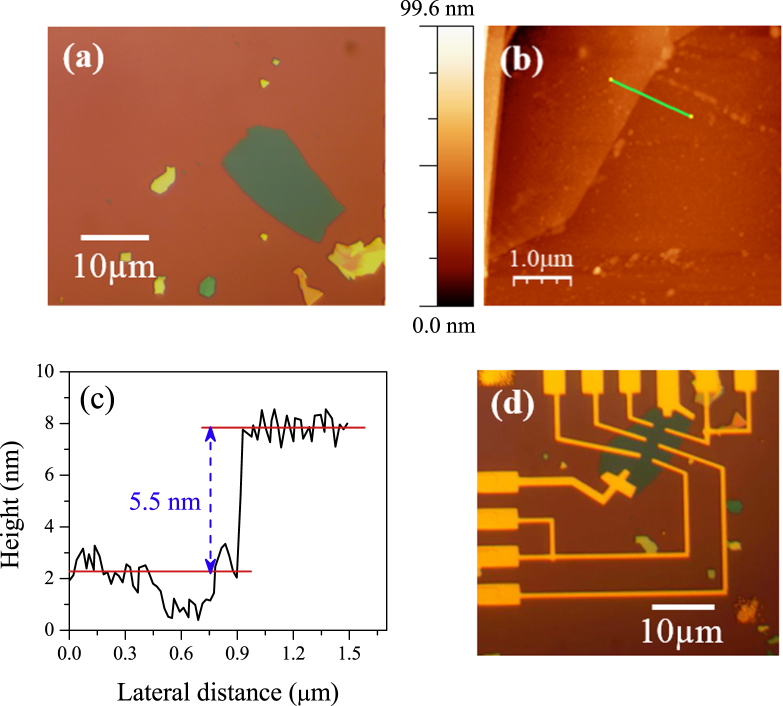
(a) The optical image of mechanically exfoliated ML MoS_2_ on Si/SiO_2_ substrate. (b) AFM image of ML MoS_2_. (c) Height profile measured along the green line in panel (b). (d) Optical image of fabricated device with source and drain electrodes of the transistors made of Cr/Au (6/80 nm).

Figure 1(a) shows the optical image of ML MoS_2_ on SiO_2_ substrate. The fabrication process started with the micromechanical exfoliation of naturally occurring crystals of molybdenite using scotch tape method. The ML MoS_2_ flakes were then transferred to a 300 nm thick SiO_2_ substrate with underlying highly p-doped silicon. ML MoS_2_ was first identified by the contrast in an optical microscope. ML MoS_2_ was further characterized by Raman spectroscopy and AFM. Figures 1(b) and (c) show the surface morphology and corresponding height profile of the ML MoS_2_ film using AFM. The thickness of MoS_2_ is approximately 5.5 nm, which indicates seven to eight layers of MoS_2_. Figure 1(d) represents the optical image of the ML MoS_2_ film with Cr/Au electrical contacts. The thickness of the Cr/Au electrical contact is 6 nm/80 nm.

Tailoring the electronic properties of semiconducting channel materials is essential for using these materials in high-performance electronic and optoelectronic devices. Several approaches have been applied to modulate the electronic properties of 2D nanomaterials by depositing dopant atoms, chemical modification by absorption of gas molecules/aromatic compounds, and by surface-induced interstitial doping [32]. In general, interstitial doping is usually difficult to control and often introduces defects, thereby reducing the mobility of host materials. Chemical modification, especially non-covalent functionalization, is one of the most effective methods to tailor the electrical properties of 2D nanomaterials. This type of chemical modification does not change the basic electronic structure and preserves the desired electronic properties of 2D nanomaterials by minimizing the damage to the lattice. In our previous reports, the electronic properties of exfoliated single-layer, bilayer and tri-layer graphene, as well as CVD-grown single-layer graphene, were tailored by PTSA molecular doping without degrading its transparency and electrical properties [30, 31].

**Figure 2.**
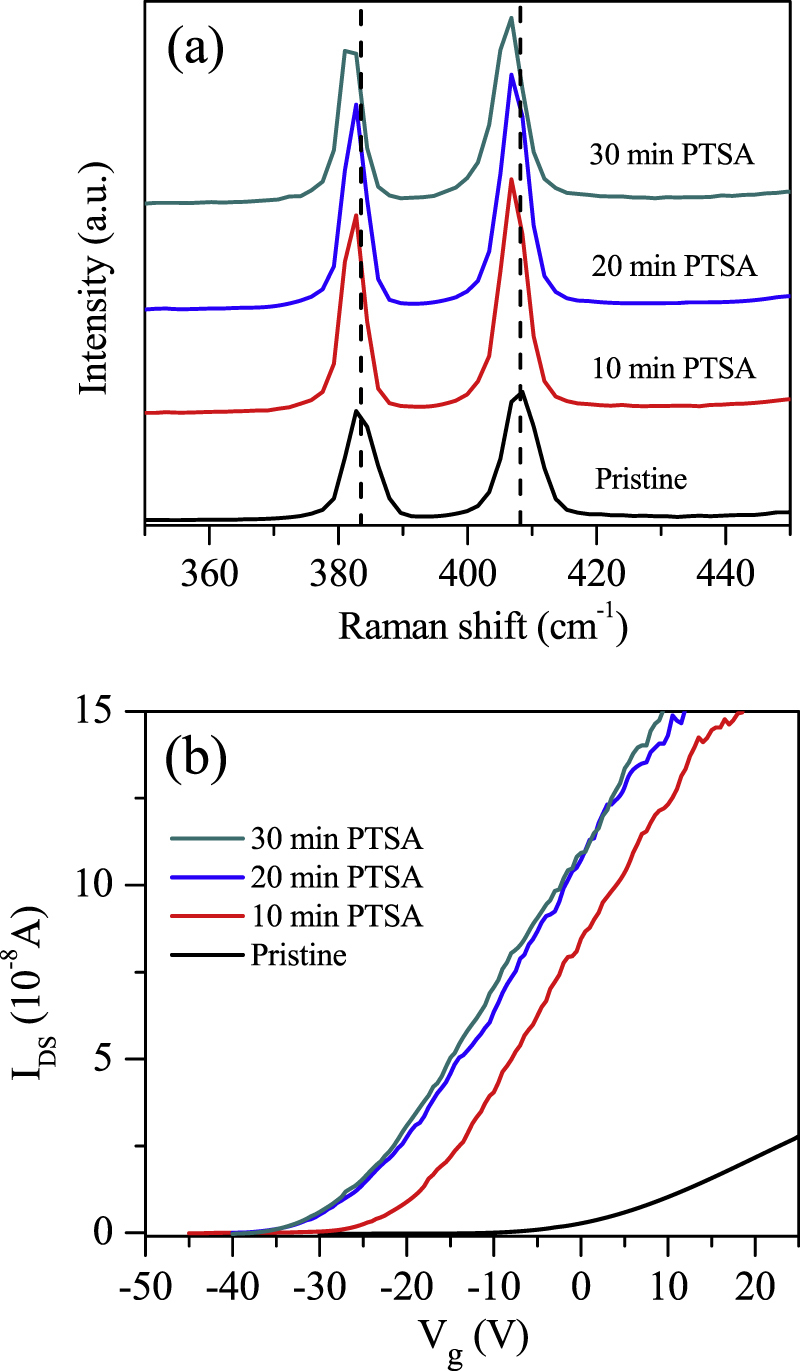
(a) The Raman spectra of ML MoS_2_ with 514 nm laser source at room temperature before and after PTSA treatment for different exposure times. (b) Drain–source current as a function of back gate voltage (*V*_g_) for ML MoS_2_ before and after PTSA treatment for different exposure times.

Figure 2(a) shows the Raman spectra of ML MoS_2_ before and after PTSA modification for different periods of time. Two characteristic peaks, E^1^_2g_ (in-plane vibration) and A_1g_ (out-of-plane vibration), appear around 384 and 408 cm^–1^, respectively. The frequency difference between these two Raman modes varies depending on the number of layers and can be easily used as a thickness indicator. The frequency difference between the Raman A_1g_ and E^1^_2g_ modes (*Δ* = A_1g_ − E^1^_2g_) is approximately 24 cm^–1^, indicating few layers. The Raman spectra of ML MoS_2_ after PTSA treatment shows downward shifting of the E^1^_2g_ and A_1g_ peak positions compared with pristine ML MoS_2_, as shown in figure 2(a). The downward shifting of the E^1^_2g_ and A_1g_ peak positions is attributed to the n-doping of MoS_2_ as previously reported by others for different systems [33, 34]. The shifting of the E^1^_2g_ and A_1g_ peaks toward low wave numbers increases with increasing PTSA exposure time. The n-doping of ML MoS_2_ is also confirmed by the electrical charge transport measurements.

Figure 2(b) displays the drain current *I*_DS_ as a function of the applied back-gate voltage *V*_g_ at a fixed drain–source voltage, *V*_DS_ = 10 mV, for pristine and PTSA-doped ML MoS_2_. All electrical characterizations of the devices were performed at room temperature in a vacuum chamber. The *I*_DS_*–V*_g_ graph reveals an n-type channel for ML MoS_2_. The *I*_DS_ shifts toward negative *V*_g_ after PTSA treatment. The shift toward the negative gate voltage increases with increasing PTSA exposure time, as shown in figure 2(b). Shifting of the threshold voltage toward the negative gate voltage reveals the n-doping in the ML MoS_2_ layers. The same trend is observed on the other devices.

**Figure 3.**
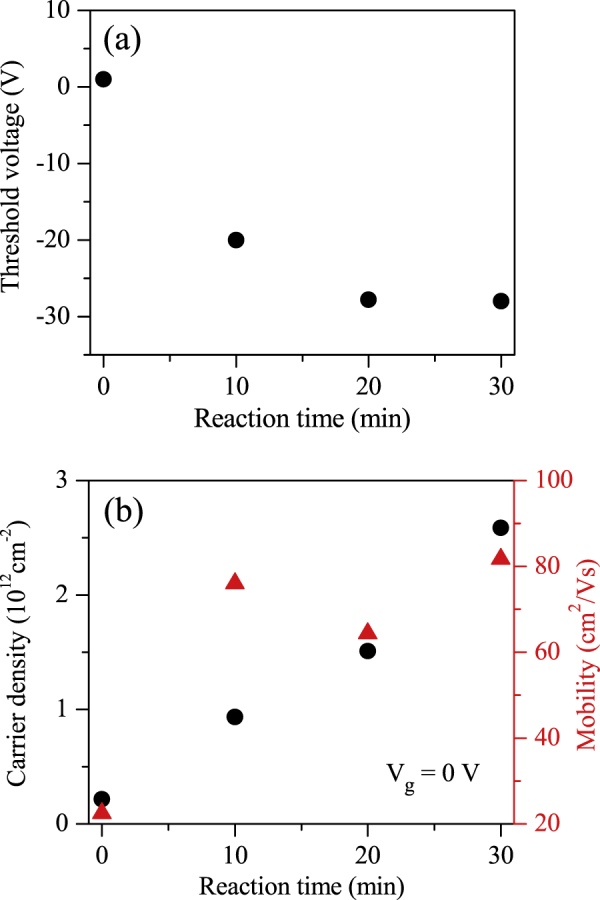
(a) Threshold voltage as a function of the PTSA exposure time of ML MoS_2_. (b) Charge carrier density at *V*_g_ = 0 V and field-effect mobility as a function of the PTSA exposure time of ML MoS_2_.

Figure 3(a) illustrates the threshold voltage as a function of PTSA exposure time. The threshold voltage shifts toward the negative gate voltage as the PTSA reaction time increases, revealing the n-doping in MoS_2_. This chemical doping turns out to be very stable and the same characteristics of the PTSA doped devices have been observed after 10 days in the ambient atmosphere.

Figure 3(b) shows the field-effect mobility of ML MoS_2_ as a function PTSA reaction time. The mobility of the samples was determined using the relation 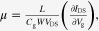 where *L* is the channel length, *W* is the channel width, 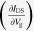 is the slope of the transfer characteristic of the device in the linear region for particular doping time, and *V*_DS_ = 0.01 V. The length and width of the fabricated device are 2.8 and 9.7 *μ*m, respectively. The gate capacitance *C*_g_ for Si/SiO_2_ is ∼115 aF *μ*m^−2^. The mobility of pristine ML MoS_2_ was measured as 22.4 cm^2^ V^−1^ s^−1^. The mobility remarkably improved after PTSA treatment, and it was found to be 84 cm^2^ V^−1^ s^−1^ after 30 min of exposure to PTSA molecules. The improvement in mobility may be due to reduction of Schottky barrier height between source/drain and MoS_2_ layers. PTSA increases the electron density in MoS_2_ channel, which changes the Fermi level of MoS_2_ and lowers the Schottky barrier height between the electrode and MoS_2_. Figure 3(b) also shows the charge carrier density (*n*) of ML MoS_2_ as a function of PTSA exposure time at *V*_g_ = 0 V. Figure 3(b) clearly shows that the charge carrier density of ML MoS_2_ significantly changed after different periods of PTSA treatment. The charge carrier densities of our ML MoS_2_ transistors were estimated using the relation 
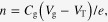
 where *e* is the elementary charge and *V*_T_ is the corresponding threshold voltage of the device at different PTSA exposure times.

The n-type doping effect by PTSA solution can be understood as follows. Mo is electropositive in nature as it belongs to 4th transition group of periodic table. The valence electronic configuration of Mo (5S^1^4d^5^) shows a capacity to accept electrons. On the other hand, 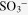 has three oxygen atoms, in which two are doubly bonded and the third one is singly bonded with S atom, so there is a resonance in the [O = S–O] bond. 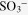 can coordinate via monodentate and bidentate moieties owing to the presence of resonance in its structure. The aromatic ring of PTSA contains two groups (CH_3_ and SO_3_), and 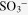 is more reactive than 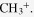 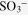 coordinates with Mo through bidentate mode and shifts electrons to s and d-orbitals of Mo. By accepting the electrons, Mo becomes electron rich and causes n-doping.

## 4. Conclusions

A simple technique to modulate the electronic properties of ML MoS_2_ by PTSA molecular doping was demonstrated. The effect of PTSA doping on the electric properties of ML MoS_2_ was investigated by Raman spectroscopy and charge transport measurements. The charge transport and Raman spectroscopy measurements revealed that PTSA molecules impose n-doping in ML MoS_2_. The threshold voltage shifted toward more negative gate voltages, thereby confirming n-doping in ML MoS_2_. The shift of the Raman peak frequencies was also analyzed as a function of reaction time. Our study demonstrated that molecular n-doping using PTSA is a feasible scheme for improving the electronic properties of MoS_2_-based devices.
